# Exploring Australian High‐Risk Foot Podiatrists' Understanding of Recurrent and Contralateral Charcot Neuroarthropathy in Individuals With Diabetes Mellitus: A Qualitative Study

**DOI:** 10.1002/jfa2.70109

**Published:** 2026-01-29

**Authors:** Keet Yeng Cheong, Karl B. Landorf, Shannon E. Munteanu, Shan M. Bergin

**Affiliations:** ^1^ Discipline of Podiatry School of Allied Health Human Services and Sport La Trobe University Victoria Australia

**Keywords:** ankle, Charcot neuroarthropathy, diabetes mellitus, foot, recurrence

## Abstract

**Introduction:**

Charcot neuroarthropathy (CN) can result in severe destruction of the foot and the ankle. Additional episodes of acute CN in the same or contralateral foot cause additional burden. This study aimed to explore Australian high‐risk foot podiatrists' understanding of recurrent and contralateral CN in individuals with diabetes.

**Methods:**

Semi‐structured online interviews were conducted. Interviews were audio‐recorded and transcribed. Thematic content analysis was used.

**Results:**

Five themes were identified in relation to recurrent and contralateral CN—two were related to potential risk factors: *patients and systems* and *addressing clinical complexity*, two were related to preventive interventions: *protection as prevention* and *knowledge and surveillance* and one was related to barriers to implementing preventive interventions: *financial and personal endurance*. The most reported risk factors were reduced adherence to management and lack of knowledge. Many preventive interventions were reported, but there was a lack of consensus on standardised care, and due to significant barriers, these interventions were not frequently used.

**Conclusion:**

This study found that participants perceived a range of potential risk factors for recurrent and contralateral CN. They also reported a variety of preventive interventions; however, due to associated barriers, these were not frequently applied. Further rigorous research is needed to develop evidence‐based guidelines.

## 1 Introduction

Charcot neuroarthropathy (CN) is an inflammatory process in individuals with peripheral neuropathy that results in destructive injury to bones, joints and soft tissues [1]. Such injuries can include fractures, joint dislocation and severe destruction of the structure of the foot and the ankle [1]. Diabetes is the most common underlying cause of neuropathy and therefore the most common precursor to CN [2]. Although it can be highly destructive, the prevalence of CN is low being estimated to range from 0.04% to 0.56% in individuals with diabetes [3, 4, 5].

Acute CN is characterised by signs of inflammation [1] and typically presents as a unilateral red, hot and swollen foot [2, 6, 7, 8, 9], though some patients (2.4% to 19.4%) present with bilateral CN with both feet affected simultaneously [10, 11, 12]. This inflammatory process can cause fractures, joint subluxation and/or joint dislocation [13]. These destructive processes can lead to permanent deformity [14] and areas of significantly increased focal pressure from footwear or ground reaction forces during standing and walking. Increased pressure is a known precursor to ulceration, which subsequently predisposes affected individuals to infection and amputation [1]. Studies have identified a 6 to 12 times increased risk of lower limb amputation in individuals who develop a foot ulcer subsequent to CN deformity [15, 16]. The 5‐year mortality rate for CN alone is estimated to be 29%, and for foot ulcer, with or without CN, it is estimated to be 30.5% [17]. Direct and indirect costs of CN and related complications are unknown in Australia; however, a 2020 study by Armstrong et al. reported direct healthcare costs for diabetes related foot disease worldwide to be approximately USD$79 billion [17]. As such, timely diagnosis and management of the acute phase of CN through offloading is imperative to minimise residual deformity [18].

Subsequent episodes of CN typically present as either a recurrence, which is defined as a subsequent episode in the same foot (but not necessarily affecting the same joints) or a new occurrence in the contralateral foot [19]. The frequency with which subsequent episodes of CN occur is difficult to estimate with any accuracy due to the lack of standardised definitions and study methodologies used. Estimates for recurrent CN range from 9% to 24.4% [19, 20, 21, 22, 23, 24]. Both recurrent and contralateral episodes of CN have been reported to lead to poorer outcomes in affected individuals and longer durations of offloading [10, 23, 25], which can be highly disruptive to their activities of daily living, roles and responsibilities [26]. Consequently, recurrent and/or contralateral episodes of CN can have a sustained negative impact on the quality of life of individuals with diabetes [12, 27].

While there is some guidance available for minimising the risk of recurrent or contralateral acute CN [1, 28], the evidence base is poor and as such, there remains wide variation in diagnosis, treatment and prevention [29]. This study aimed to explore Australian high‐risk foot podiatrists' understanding of recurrent or contralateral CN in individuals with diabetes.

## 2 Methods

### 2.1 Ethical Considerations

Ethics approval for the study was obtained from La Trobe University Human Research Ethics Committee (HEC23287). This study is reported according to the Standards for Reporting Qualitative Research (SRQR) [30].

### 2.2 Research Team

The research team comprised a graduate researcher (K.Y.C.) who attended a qualitative research training course prior to the study and three researchers (K.B.L., S.E.M. and S.M.B.) with experience in quantitative and qualitative research relating to the foot and the ankle, including semi‐structured interviews and thematic analysis. Both the first author (K.Y.C.) and the senior author (S.M.B.) have also worked extensively with individuals with diabetes‐related foot disease, including the assessment and treatment of CN.

### 2.3 Study Design, Sampling and Recruitment

This study was a qualitative study that used semi‐structured interviews. An inductive qualitative approach was used for this study with the inclusion of some basic frequency counts of responses. Accordingly, the study used thematic content analysis to explore the data generated from the interviews. Interviews were conducted with registered podiatrists in Australia who routinely manage CN in individuals with diabetes. Purposive sampling was used to ensure recruited participants had the requisite clinical knowledge and experience and were representative of a range of clinical contexts. Potential participants were identified via well known and easily identified clinical networks, including established high‐risk foot services (i.e., in major metropolitan and regional city teaching hospitals) and the Advanced Practicing Podiatrists—High‐Risk Foot Group (a professional special interest group aimed at podiatrists practising in high‐risk foot services).

A study advertisement was circulated via email. Interested individuals were invited to contact the research team at which time a participant information statement and consent form was provided. On confirmation of their eligibility to participate in the study, a date and time for an online interview was confirmed. At the time each participant joined their online interview, a link to a REDCap (Research Electronic Data Capture) online form was forwarded via email. Each participant used this form to provide electronic consent prior to proceeding with the interview.

Previous qualitative studies in related fields of research indicated that an initial sample size estimate of approximately 16 participants was appropriate [31, 32]. However, in keeping with accepted qualitative study methodology, we adopted the approach of ceasing recruitment once data saturation was reached [33].

### 2.4 Data Collection and Management

Participants were de‐identified for the purposes of data analysis. Information relating to participant characteristics including gender, ethnicity, year of podiatry qualification, years of work as a podiatrist, years of work in a high‐risk foot service, primary clinical work setting, equivalent full‐time (EFT) workload, and geographical location of primary clinical practice was collected using REDCap, which was hosted at La Trobe University [34, 35]. Interviews were conducted between November 2023 and April 2024 using an online platform (Zoom Video Communications Inc., San Jose, California, United States). The primary investigator (K.Y.C.) led the interview process with a second investigator (S.M.B. or K.B.L.) present for the purposes of taking relevant notes and identifying additional areas for exploration during the interviews. Interviews were of approximately 60 min in duration and consisted of open‐ended questions supported by an interview guide [36]. The questions included in the interview guide can be found in Supporting Information S1.

Interviews were audio‐recorded using the recording function on Zoom. Audio recordings were then transcribed by an independent, confidential transcription service. Transcribed data were first verified by K.Y.C. and then to ensure rigour, participants reviewed a copy of their transcribed interviews for accuracy as part of a member checking process.

### 2.5 Data Analysis

The first three transcripts were independently reviewed by two investigators (K.Y.C. and S.M.B.). Each investigator independently identified significant ideas, concepts or elements [32] within each transcript and then grouped these thematically. The researchers then conferred to reach agreement on included themes, subsequently applying codes that could be tagged to transcribed data [37] using NVivo (Lumivero LLC, Denver, United States). K.Y.C. then reviewed all remaining transcripts and applied the agreed coding framework.

## 3 Results

Twelve podiatrists participated in this study; participant characteristics are presented in Table 1.

**TABLE 1:** Participant characteristics (*n =* 12).

Characteristic	Frequency or mean ± SD
Ethnicity, *n* (%)	Australian, *n* = 4 (33%)
	British, *n* = 1 (8%)
	Chinese, *n* = 1 (8%)
	English, *n* = 2 (17%)
	French, *n* = 1 (8%)
	Indian, *n* = 1 (8%)
	Irish, *n* = 1 (8%)
	Scottish, *n* = 1 (8%)
Year of podiatry qualification	0–5 years ago, *n* = 1 (8%)
	6–10 years ago, *n* = 3 (25%)
	11–15 years ago, *n* = 2 (17%)
	> 15 years ago, *n* = 6 (50%)
Years of work as a podiatrist (mean ± SD)	13.5 ± 6.6
Years of work in a high‐risk foot service (mean ± SD)	9.8 ± 5.5
Primary clinical work setting (present time), *n* (%)	Hospital‐based service, *n* = 11 (92%)
	Community‐based service, *n* = 1 (8%)
EFT (mean ± SD)	0.9 ± 0.2
Geographical location of primary clinical practice, *n* (%)	Major cities of Australia, *n* = 8 (67%)
	Inner regional Australia, *n* = 2 (17%)
	Outer regional Australia, *n* = 1 (8%)
	Remote Australia, *n* = 1 (8%)
	Very remote Australia, *n* = 0

Abbreviations: EFT, equivalent full time; SD, standard deviation.

### 3.1 Defining Recurrent and Contralateral CN

Most participants (*n* = 11) were able to define recurrent and contralateral CN and demonstrated a comprehensive understanding of both. The remaining participant was unsure of the accepted definitions for each condition; however, they agreed that the definitions provided during the interview aligned with their own understanding of how these conditions presented. In addition, most participants (*n* = 11) had encountered both recurrent and contralateral acute CN in their clinical practice. The remaining participant advised that though their caseload included patients with CN, their geographical location meant that their exposure was limited.

### 3.2 Potential Risk Factors for Development of Recurrent and Contralateral CN

Participants reported a wide range of potential risk factors—43 in total—for the development of recurrent and contralateral CN. Twenty‐two potential risk factors were reported for the development of recurrent CN, and 21 potential risk factors were reported for the development of contralateral CN. Of these, 15 were common to both complications (Table 2). Although participants reported a range of potential risk factors, they were less clear about the strength of available evidence to support a causal relationship between each risk factor and the onset of recurrent or contralateral CN.

**TABLE 2:** Commonly reported potential risk factors for the development of recurrent and contralateral CN.

Risk factors	Recurrent (*n*)	Contralateral (*n*)
Reduced adherence to management	11	9
Poor knowledge of CN in healthcare community	8	8
Trauma	6	4
Reduced access to services and/or resources	5	5
Neuropathy	5	5
Suboptimal glycaemic control	5	4
Treatment fatigue	5	1
Excessive or premature loading of the affected or the contralateral limb	4	8

Abbreviation: CN, Charcot neuroarthropathy.

#### 3.2.1 Theme 1: Patients and Systems

Thematic analysis identified that potential risk factors for recurrent or contralateral CN could be attributed to either patient‐ or system‐related factors. Patient‐related factors were associated with management of initial or subsequent CN episodes, patient understanding of the importance of treatments such as prolonged offloading, and the presence of known precursors to CN such as neuropathy.

Commonly reported patient‐related factors common to both conditions included reduced adherence to CN management plans, trauma, presence of neuropathy, and suboptimal glycaemic control. Reduced adherence to management plans was perceived by participants to be associated with a lack of insight by patients regarding their condition and the role of offloading in treatment. Other reported risk factors included treatment fatigue associated with long‐term use of offloading devices and an eagerness to resume disrupted roles and responsibilities. Specific to recurrent CN, participants reported that rapid loading of a deconditioned or osteopenic Charcot limb after prolonged immobilisation was a potential risk factor. Specific to contralateral CN, participants reported that overloading of the unaffected contralateral foot during an initial CN episode was a potential risk factor, and this risk could persist after acute CN of the other foot had resolved due to the presence of residual deformity and changes to lower limb and foot mechanics. The following quotations from participants highlight the issues outlined above.

… Why has it reactivated? (…) is it possibly the patient isn’t wearing their device, they’re not compliant, but then if they’re not compliant, understanding why, why they’re not compliant? Maybe because they don’t like it for whatever reason, or maybe because it doesn’t meet their goals of daily activity, so understanding that ….

(Participant 07)

Some of the factors that could increase the risk or rate of recurrent Charcot? I guess—well, we know that they’ve got the predisposing factor of neuropathy already in the foot.

(Participant 10)

… I think one of the other issues we had and again I have absolutely no data on this but a perception is that I think sometimes we are too quick to take someone out of an appropriate offloading device, whether that’s because of very rarely clinical—like clinician fatigue, but more so the patient is sick of being in a cast. Wants to progress to the next phase or might have some pressures from work or family commitments to come out of that.

(Participant 02)

… when you’re instructed to limit your weightbearing on one foot, then they will most likely transfer a lot of the weight to the … normal foot or the unaffected foot …

(Participant 04)

System‐related factors included poor knowledge of CN within the wider medical and healthcare community, reduced access to healthcare services due to extensive geographical distances, and reduced access to resources for assessment, diagnosis, and management of CN.

Poor knowledge about CN in the wider medical and healthcare community had important implications for timely diagnosis and management of acute CN, which was identified as being a particular issue in remote communities due to limited healthcare staffing and accessibility to specialist services.

… maybe the lack of knowledge about Charcot in the wider health community as well. … if more people, more disciplines, more clinics knew about Charcot, they’d be monitoring and hopefully picking up early if things go wrong. … they can really promote the preventative strategies that we already have in place, which is checking regularly, protective footwear, et cetera.

(Participant 10)

So let’s say, you did have a Charcot, and you’re out in community, and you don’t have any ways to come to the clinic and you have to walk two hours, and you might not wear your medical grade footwear, then obviously, your risk of having a Charcot to trigger again is going to be through the roof. So there’s that complexity as well, that we might not necessarily find when you’re working in the more urban settings.

(Participant 06)

The lack of knowledge about CN may also result in the prescription of interventions by healthcare providers that may increase the risk of recurrent and/or contralateral CN occurring. One such example would be the implementation of strenuous physical activities shortly after the resolution of acute CN, potentially overloading the limb initially affected by CN or the contralateral limb, resulting in a recurrent or contralateral episode.

… more education to other medical colleagues, whether that’s someone—a medical doctor, physio, osteo, personal trainers, I think this is really important. Like these people, sometimes if they’re really motivated, they will engage with a personal trainer to lose weight after that (acute CN episode). Them being aware of the risks of loading up that other limb is really important as well.

(Participant 02)

#### 3.2.2 Theme 2: Addressing Clinical Complexity

Additional risk for development of recurrent or contralateral CN was attributed to the overall complexity of CN and knowledge gaps that still exist in terms of assessment, diagnosis and management of CN. Participants described limitations associated with the overreliance on skin temperature measurements for the clinical diagnosis and monitoring of acute CN and lack of standardised temperature monitoring protocols. Participants were also challenged by the complexities associated with management of acute bilateral CN presentations, clinical indicators to accurately identify remission of acute CN, and diagnosis and management of atypical presentations, such as the presence of pain and peripheral arterial disease.

We’ve had so many odd Charcots … we’ve had people who are not completely neuropathic, we’ve had a Charcot in a lower leg … the more I learn about Charcot, the more I don’t know about Charcot, I think is basically what I’ve been discovering.

(Participant 01)

… we had to do lots of streamlining around measuring of temperatures, because I’ve noticed from one podiatrist to another, we were not using the Dermatemp the same way. I had to do work instructions, so that everybody was the same—and we had to also be careful as to mirroring the time when the cast’s been coming off, so within 15 minutes, and same with not washing the feet, because we’ve noticed that that’s been impacting on (temperatures).

(Participant 06)

### 3.3 Preventive Interventions to Reduce the Likelihood of Recurrent or Contralateral CN

Participants reported 19 preventive interventions to reduce the risk of recurrent CN and 23 preventive interventions to reduce the risk of contralateral CN occurring. Of these, 12 were common to both presentations (Table 3). The most prevalent themes as they relate to prevention were management of load through the feet, clinician and patient education, and long‐term monitoring of at‐risk patients.

**TABLE 3:** Commonly reported preventive interventions to reduce risk of recurrent and contralateral CN.

Preventive interventions	Recurrent (*n*)	Contralateral (*n*)
Protection and offloading	10	11
Provision of patient/clinician education	10	9
Regular monitoring and follow‐up	8	6
Optimisation of glycaemic control	4	3
Rehabilitation following prolonged immobilisation	3	4
Use of skin temperatures for ongoing monitoring	3	2
Standardised and gradual transition to long‐term offloading	3	2
Psychology support	2	2

Abbreviation: CN, Charcot neuroarthropathy.

#### 3.3.1 Theme 3: Protection as Prevention

The primary aims of preventive interventions were protection and offloading of vulnerable feet through use of off‐the‐shelf and medical grade footwear and offloading devices including orthoses. There were points of difference depending on whether the prevention was aimed at recurrent or contralateral CN; however, protective interventions were commonly employed by most of the participants.

For recurrent CN, protection and offloading were attempted through use of appropriate off‐the‐shelf footwear including sports shoes, use of orthopaedic footwear, prescription of prefabricated or custom‐made medical grade footwear, prescription of custom‐made or prefabricated foot orthoses or use of removable knee‐high offloading devices.

… all of our patients that have had Charcot should have custom insoles to support their new foot structure, and generally extra depth width footwear, and that usually involves having a rocker sole as well to accommodate the deformity.

(Participant 05)

To prevent onset of contralateral CN, participants reported they applied interventions including prefabricated or custom foot orthoses to the unaffected foot. Heel raises and shoe balancers were also used to reduce any limb length discrepancy resulting from devices used to manage the affected lower limb.

One of my biggest gripes … is when we put an orthotic in the affected Charcot foot and we don’t prescribe an orthotic for the other foot. I think we just missed a massive opportunity there to again—we don’t have evidence on it but if you even out those plantar pressures with—look at getting that other foot to function in the best way that it possibly can, I think that has a massive impact (on preventing contralateral acute CN).

(Participant 02)

#### 3.3.2 Theme 4: Knowledge and Surveillance

Provision of education, whether for the patient and/or clinician, was a common preventive intervention among participants. Patient‐specific education included safe foot care and footwear practices, whereas both patient and health professional education included recognition of signs and symptoms of acute CN and escalation pathways when there is cause for concern.

So part of my discharge plan personally is that I advise them to be in supportive footwear and insoles 100% of their weightbearing time, and even when they go to the bathroom, I advise them to wear the shoes into the bathroom and then take them off for their shower.

(Participant 04)

We educate the patient as to what to be aware of—pain, colour changes, foot structure changes, how to look after their foot, the importance of wearing the offloading both in the house and outside of the house. The patient knows how to escalate their care directly to us, or to emergency if outside of our hours.

(Participant 07)

Regular monitoring and follow‐up appointments were reported as important for the prevention of recurrent or contralateral CN and were widely used. Participants indicated that although podiatrists were commonly involved in ongoing review and management, other healthcare providers were also utilised depending on service delivery models and accessibility to specialised services.

So definitely I think the regular follow‐ups with a specialist. So in our district, I would say a podiatrist definitely because we’ve got our community clinics. But I guess it doesn’t have to just be a podiatrist. It could be a nurse or another appropriate discipline as well.

(Participant 10)

A preventive intervention that was reported as being potentially useful but not widely used for either recurrent or contralateral CN is skin temperature monitoring as part of routine monitoring by podiatrists or by patients in their home. This appeared to relate to uncertainty regarding its usefulness as part of long‐term surveillance.

I can’t say that we routinely measure temperatures on the other limb for patients who have had a Charcot. I think something that might be good to come out of all this is that if we look at the numbers and we work out that there is actually a risk of Charcot in the other foot, maybe we should be measuring temperatures routinely, but I can’t say that we do that.

(Participant 12)

### 3.4 Barriers to Implementing Preventive Interventions for the Development of Recurrent and Contralateral CN

Participants reported a broad range of interventions that could potentially prevent the onset of recurrent and contralateral CN. Many of these were routinely used by participants; however, significant barriers were also identified that made it challenging to apply these interventions to all patients (Table 4).

**TABLE 4:** Commonly reported barriers to the implementation of preventive interventions for recurrent and contralateral CN.

Barriers to implementation of preventive interventions	Recurrent (*n*)	Contralateral (*n*)
Patient beliefs and preferences for care	12	10
Lack of knowledge of CN in healthcare community	8	8
Financial and/or funding issues	8	8
Poor evidence base for CN	6	5
Treatment fatigue	5	1
Competing roles and responsibilities	5	3
Availability of services and resources outside of metropolitan areas	5	5

Abbreviation: CN, Charcot neuroarthropathy.

#### 3.4.1 Theme 5: Financial and Personal Endurance

The cost associated with the provision of long‐term offloading devices, such as medical grade footwear, orthoses and Charcot restraint orthotic walkers, was identified as a barrier for both complications. The cost of offloading devices can be prohibitive for some patients. Although funding schemes in Australia are available to those who are most in need (e.g., the Statewide Equipment Program or internal hospital funding support), significant out‐of‐pocket expenses are not uncommon, so patients either cannot access these devices and/or they limit using them for fear of losing, damaging or wearing them out. Further, participants described the process of applying for available funding as complicated, tedious and time‐consuming with wait times for disbursement of funds often prolonged.

And so you know, you will give patients custom insoles and shoes and it costs a thousand dollars, and they might still present in their (regular) shoes a few weeks later, because they’re trying to keep those good shoes new and good, whereas we want them wearing them and using them. You see it a lot, unfortunately.

(Participant 05)

Even applying for (name of funding stream) is hard work just for the clinician. It takes up a lot of time just to do the application and then get online and submit it and then there’s always a problem with the funding application. It takes up like an hour of your time just to submit it, and then a patient has to wait six months before they get the funding ….

(Participant 05)

The beliefs and preferences of individuals with CN towards care was reported as a further barrier to the implementation of preventive interventions for the development of recurrent and contralateral CN. Participants shared that not all affected individuals were willing or able to use the footwear or other offloading devices that had been recommended. Patient fatigue associated with long‐term immobilisation and prolonged disruption to roles and responsibilities were also considered to play a role.

Barriers are definitely patients not wanting to wear the boots because the boots are heavy, they’re uncomfortable.

(Participant 11)

It may also be a patient's perceptions of footwear that can be a barrier. Patients may not want to wear custom or medical‐grade footwear. They may like walking barefoot, they might like going to the beach and not wearing shoes.

(Participant 12)

… if there’s a recurrence of Charcot a year or more down the track, is—I think that they really struggle with that and they also see their lives really changing drastically, because it’s the second time round they realise they can’t work, they realise they can’t manage at home as well. They realise financially they’re struggling. So you know they—both physically, mentally, everything seems to deteriorate quite rapidly from then, if it hasn’t already.

(Participant 05)

… other barrier for prevention is also from the patient, in terms of they’ve potentially been in an offloading knee‐high device for a fair few months, potentially close to a year or over a year we’ve had some patients in offloading devices. So sometimes I think their level of commitment to being in a restrictive—say, they might need to go into an ankle high boot, for example, because of the level of mobility in their resulting foot. I think that can be a barrier for prevention of a recurrent Charcot, because they’re just a bit over it and don’t really want to be in a restrictive shoe anymore.

(Participant 10)

## 4 Discussion

The findings from this study indicate that participants had a good understanding of recurrent and contralateral CN as complications after an initial CN episode and they reported a wide range of potential risk factors. The reporting of 15 different potential risk factors common to both recurrent and contralateral CN underscores the perceived multifactorial nature of their development and the complexities clinicians face when assessing and managing affected patients. Participant uncertainty regarding the strength of available evidence to support the potential risk factors they reported highlights gaps in our current understanding of CN overall. Accordingly, there is an urgent need for further research to establish clear causal relationships.

Both patient‐ and system‐related factors were reported by participants in our study as potential risk factors for the onset of recurrent and contralateral CN. Patient‐specific factors, such as trauma, presence of neuropathy and suboptimal glycaemic control, align with existing literature related to risk factors for other diabetes‐related foot complications including ulceration [38, 39, 40]. This highlights that there may be a need for education of patients with diabetes to include these as potential risk factors for CN and for patients with an initial episode of CN to be informed about the potential for development of recurrent and contralateral CN. However, further research is needed to determine if these are, indeed, true risk factors. Patient education related to signs and symptoms of CN is also essential to support clinicians and patients to engage in vigilant monitoring and early intervention wherever possible.

Participants also reported that poor patient adherence to treatment protocols and early return to normal activity after an initial CN episode are potential risk factors for development of recurrent and contralateral CN. Interestingly, participants also identified adherence as a barrier to implementing some preventive interventions. Treatment fatigue, disruption to activities of daily living, restricted or lost employment, and negative impacts on mental and emotional wellbeing are all well documented consequences of chronic conditions including diabetes, diabetes‐related foot ulceration and CN [26, 27, 41, 42, 43, 44]. For those patients who have already experienced extended treatment and immobilisation for an initial CN episode, it is not surprising that their adherence is challenged with subsequent episodes related to recurrent or contralateral CN.

System‐related factors included poor understanding and awareness of CN overall, leading to lack of understanding of potential risks associated with recurrent and contralateral CN. Geographical location outside of major metropolitan areas was another system‐related factor and was reported to be associated with reduced access to specialist services and extended wait times for appointments. These findings further highlight the disparities that exist in terms of healthcare access and the need for targeted solutions to facilitate improved outcomes for individuals at risk of recurrent and contralateral CN living in underserviced areas [45].

In consideration of the potential risk factors reported, participants were asked about preventive interventions that may be implemented to reduce the risk of recurrent and contralateral CN and how readily they applied these interventions. Prevention of other diabetes‐related foot complications such as neuropathy, peripheral arterial disease and ulceration are well defined in the literature and current clinical guidelines [46, 47, 48, 49, 50]. This is made easier by a clear understanding of the mechanisms that predispose patients to the development of these complications, that is, there is robust evidence. A 2023 paper by Schaper et al. clearly articulates known risk factors for foot ulceration including loss of protective sensation and/or presence of peripheral arterial disease, foot deformity and some precipitating event such as trauma or pressure [40]. With risk factors and causal relationships established, the identification of preventive interventions is made easier [40, 46]. In contrast, there is a lack of evidence for risk factors for recurrent and contralateral CN [51], which likely explains why participants in this study reported 42 potential preventive interventions for recurrent and contralateral CN combined, with 12 common to both complications. The most reported preventive interventions were management of load through foot, patient, and clinician education and routine monitoring and surveillance. Despite the abundance of potential preventive interventions nominated, the majority were not routinely implemented due to perceived barriers or challenges associated with doing so.

Participants shared their experiences of clinician and patient frustration and fatigue with extended periods of treatment and immobilisation often leading to reduced patient adherence to treatment protocols. These findings are not unexpected if the 2022 qualitative study by Gooday et al. is considered, where 14 patients living with CN identified feelings of loss and isolation, a significant disruption to roles, responsibilities and relationships, and guilt associated with needing greater support and for developing CN in the first instance [26]. Participants in the study by Gooday et al. also reported living with pain associated with use of offloading devices, low mood and poor self‐esteem [26]. This adds a significant challenge to clinicians seeking to implement preventive and/or treatment protocols that optimise clinical outcomes while being acceptable to patients presenting with recurrent or contralateral CN who have already experienced significant disruption related to an initial CN episode.

Protection through offloading vulnerable feet was reported as an important preventive intervention. However, the high cost and administrative load associated with procurement, manufacturing and maintenance of offloading devices, including medical grade footwear, discouraged participants and the patients they treat from their routine use. The 2018 Diabetic Foot Australia guideline relating to footwear for people with diabetes recommends the use of medical grade footwear and custom‐made orthoses to manage deformity and ulcer risk, but it also acknowledges the complexity in securing funding for these interventions [52]. Patients who are required to self‐fund for long‐term offloading devices are typically reluctant to do so as this exacerbates existing financial hardship [27, 53]. Fejfarová et al. (2014) reported that significantly more individuals with diabetes‐related foot ulceration and CN received disability pensions, and significantly less were in full‐time employment than those without foot complications, reducing their capacity to self‐fund [27].

### 4.1 Implications for Clinical Practice and Future Research

An important finding from this study is the ongoing challenge clinicians face in the absence of robust evidence related to the pathophysiology, assessment and management of CN, and the onset of recurrent and contralateral CN as complicating factors. Study participants identified that the clinical complexity of CN, as an initial episode or as a recurrent or contralateral episode, made identification and management of risk factors extremely difficult. For example, in their early stages, CN and soft tissue and/or bone infection can present similarly, which adds uncertainty to the diagnosis and management [28]. Participants also reported that there is no clear guidance relating to factors that impacted their clinical care (e.g., indicators of the transition from acute to chronic CN and use of temperature measurements for treatment and routine monitoring). There is an urgent need, therefore, for high‐quality research that investigates several facets related to assessment and management of CN, including recurrent and contralateral episodes, to support clinicians to provide standardised and effective clinical care. In addition, further investigation is needed in relation to the challenges of managing the negative physical and emotional impacts of sustained and disruptive CN treatment. Studies are also needed that document the cost imposed on patients through reduced employment, increased reliance on welfare, and lack of access to funding for essential treatment, all of which influence patient adherence and treatment outcomes.

### 4.2 Strengths and Limitations

This study has several strengths. Firstly, it used an online platform to conduct interviews, in turn leading to more generalisable findings as this enabled access to podiatrists working throughout Australia. Secondly, methodological rigour was supported by the presence of two researchers to fulfil the respective roles of interviewer and note taking during each interview, a member checking process that enabled participants to review their transcribed interviews for accuracy, and the development of a robust coding framework by two researchers. Thirdly, it used a purposive sampling technique for participant recruitment, which focused on the most relevant population of Australian podiatrists (i.e., high‐risk foot podiatrists) for this research.

This study should also be interpreted in the context of its limitations. Firstly, the sample did not include any podiatrists outside of the high‐risk foot and public health settings and as such, its findings cannot be generalised to podiatrists who do not work in these settings. Secondly, we did not have proportional representation of participants from all states in Australia (i.e., we had no representation from Western Australia, South Australia, Tasmania, Australian Capital Territory, or Northern Territory), although we do not believe that this would have a significant effect on findings. Thirdly, it is important to acknowledge the subjectivity and background of the researcher in reflexive thematic analysis [54] and content analysis [55], that is, the interpretation of the data and generation of themes are potentially influenced by the prior experience of study investigators. Therefore, it is possible that the study data would be interpreted differently by other researchers [54, 55].

## 5 Conclusion

This study found that Australian high‐risk foot podiatrists reported a range of potential risk factors for the development of recurrent and contralateral CN in individuals with diabetes. Participants also reported a variety of preventive interventions for both complications; however, due to associated barriers, only a few were frequently used. This is not surprising given the poor evidence base relating to these issues. Given the potential for recurrent and contralateral acute CN to result in poor clinical outcomes, further research is needed for the development of evidence‐based clinical guidelines for the prevention of recurrent and contralateral CN.

## Author Contributions

**Keet Yeng Cheong:** conceptualisation, formal analysis, investigation, methodology, writing – original draft, writing – review and editing. **Karl B. Landorf:** conceptualisation, investigation, writing – review and editing. **Shannon E. Munteanu:** conceptualisation, writing – review and editing. **Shan M. Bergin:** conceptualisation, formal analysis, investigation, methodology, writing – review and editing.

## Funding

Keet Yeng Cheong is a Master of Applied Science candidate at La Trobe University and has received funding for this research project from the Higher Degree by Research (HDR) Support Grant provided by the School of Allied Health, Human Services and Sport.

## Ethics Statement

Ethics approval for the study was provided by La Trobe University Human Research Ethics Committee (HEC23287).

## Consent

The authors have nothing to report.

## Conflicts of Interest

K.B.L. is an Emeritus Editor, S.E.M. is a previous Deputy Editor, and S.M.B. is an Associate Editor of the *Journal of Foot and Ankle Research*.

## Supporting information


Supporting Information S1

## Data Availability

Data from this study are not available for other research projects as not all participants gave written consent for their data to be shared publicly.
