# Supplementary material for: Exploring Australian High‐Risk Foot Podiatrists' Understanding of Recurrent and Contralateral Charcot Neuroarthropathy in Individuals With Diabetes Mellitus: A Qualitative Study
Source: J Foot Ankle Res. 2026 Jan 29;19(1):e70109. doi: 10.1002/jfa2.70109 (PMC12856057; doi:10.1002/jfa2.70109)
Supplement: Supplementary file 1 — Supporting Information S1 [file JFA2-19-e70109-s001.pdf]

SUPPLEMENTARY FILE

SUPPLEMENTARY FILE 1

Interview questions

- What is your definition of recurrent acute Charcot neuroarthropathy?
- Can you tell me about your clinical experience with recurrent acute Charcot neuroarthropathy in patients with diabetes mellitus?
- Can you describe some of the factors that you think increase the risk of recurrent acute Charcot neuroarthropathy in this population?
- Why do you think they may increase the risk of recurrent acute Charcot neuroarthropathy?
- Can you describe any measures that may be implemented to address these factors?
- How did you come to know about these measures?
- Can you tell me about any measures that have been put in place by your team to prevent recurrent acute Charcot neuroarthropathy?
- What are the barriers to implementing strategies to reduce risk of recurrent acute Charcot neuroarthropathy?
- Can you start by sharing with me your definition of contralateral Charcot neuroarthropathy?
- Can you tell me about your clinical experience with contralateral Charcot neuroarthropathy in patients with diabetes mellitus?

- Can you describe some of the factors that you think increase the risk of contralateral Charcot neuroarthropathy in this population?
- Why do you think they may increase the risk of contralateral Charcot neuroarthropathy?
- Can you describe any measures that may be implemented to address these factors?
- How did you come to know about these measures?
- Can you tell me about any measures that have been put in place by your team to prevent contralateral acute Charcot neuroarthropathy?
- What are the barriers to implementing strategies to reduce the risk of developing contralateral acute Charcot neuroarthropathy?
